# Complement Receptor 1 Is a Sialic Acid-Independent Erythrocyte Receptor of *Plasmodium falciparum*

**DOI:** 10.1371/journal.ppat.1000968

**Published:** 2010-06-17

**Authors:** Carmenza Spadafora, Gordon A. Awandare, Karen M. Kopydlowski, Jozsef Czege, J. Kathleen Moch, Robert W. Finberg, George C. Tsokos, José A. Stoute

**Affiliations:** 1 Department of Medicine, the Uniformed Services University of the Health Sciences, Bethesda, Maryland, United States of America; 2 Department of Cellular Injury, Division of Military Casualty Research, the Walter Reed Army Institute of Research, Silver Spring, Maryland, United States of America; 3 Instituto de Investigaciones Científicas y Servicios de Alta Tecnología-AIP (INDICASAT-AIP), Ciudad del Saber, Clayton, Panamá; 4 Division of Malaria Vaccine Development, the Walter Reed Army Institute of Research, Silver Spring, Maryland, United States of America; 5 Department of Parasitology, Division of Experimental Therapeutics, the Walter Reed Army Institute of Research, Silver Spring, Maryland, United States of America; 6 Biomedical Instrumentation Center, the Uniformed Services University of the Health Sciences, Bethesda, Maryland, United States of America; 7 Department of Medicine, University of Massachusetts Medical School, Worcester, Massachusetts, United States of America; 8 Department of Medicine, Beth Israel Deaconess Medical Center, Harvard Medical School, Boston, Massachusetts, United States of America; 9 Department of Medicine, Division of Infectious Diseases and Epidemiology, Pennsylvania State University College of Medicine, Hershey, Pennsylvania, United States of America; Seattle Biomedical Research Institute, United States of America

## Abstract

*Plasmodium falciparum* is a highly lethal malaria parasite of humans. A major portion of its life cycle is dedicated to invading and multiplying inside erythrocytes. The molecular mechanisms of erythrocyte invasion are incompletely understood. *P. falciparum* depends heavily on sialic acid present on glycophorins to invade erythrocytes. However, a significant proportion of laboratory and field isolates are also able to invade erythrocytes in a sialic acid-independent manner. The identity of the erythrocyte sialic acid-independent receptor has been a mystery for decades. We report here that the complement receptor 1 (CR1) is a sialic acid-independent receptor for the invasion of erythrocytes by *P. falciparum*. We show that soluble CR1 (sCR1) as well as polyclonal and monoclonal antibodies against CR1 inhibit sialic acid-independent invasion in a variety of laboratory strains and wild isolates, and that merozoites interact directly with CR1 on the erythrocyte surface and with sCR1-coated microspheres. Also, the invasion of neuraminidase-treated erythrocytes correlates with the level of CR1 expression. Finally, both sialic acid-independent and dependent strains invade CR1 transgenic mouse erythrocytes preferentially over wild-type erythrocytes but invasion by the latter is more sensitive to neuraminidase. These results suggest that both sialic acid-dependent and independent strains interact with CR1 in the normal red cell during the invasion process. However, only sialic acid-independent strains can do so without the presence of glycophorin sialic acid. Our results close a longstanding and important gap in the understanding of the mechanism of erythrocyte invasion by *P. falciparum* that will eventually make possible the development of an effective blood stage vaccine.

## Author Summary

*Plasmodium falciparum* malaria is a blood parasite that lives for the most part inside red cells. It is responsible for the death of 1-2 million people every year. The mechanisms by which the parasite invades red cells are complex and not completely understood. For many years it has been known that proteins called glycophorins are used by the parasite to gain entry into the red cell. However, the existence of another protein that allows entry independent of glycophorins has been suspected for nearly as long. The identity of the alternative protein has been a mystery difficult to solve. In this article we present strong evidence that the alternative protein is the complement receptor 1. The complement receptor 1 is a well-studied protein that is known to be important in protecting red cells from attack by the host immune system as well as suspected of having other roles in the development of malaria complications. The recognition of the additional role of complement receptor 1 in red cell invasion will allow the definitive identification of malaria proteins that interact with it and that could be used in a future vaccine cocktail to block red cell invasion.

## Introduction

The erythrocyte invasion mechanisms of *P. falciparum* are varied and complex. Erythrocytes are rich in surface glycophorins which contain sialic acid. Earlier studies demonstrated that invasion of erythrocytes could be inhibited by treatment of erythrocytes with neuraminidase, which removes sialic acid, or blocked by purified glycophorin A [1]–[3]. In addition, erythrocytes genetically deficient in glycophorin A [En(a-)], glycophorin B (S-s-U-), or sialic acid (Tn) showed reduced invasion compared to normal cells [1], [4]. These studies suggested that sialic acid and the peptide backbones of glycophorin A and B play a role in the invasion of erythrocytes by *P. falciparum*. Glycophorin C was later added to this list [5]. However, some malaria strains exhibit sialic acid-independent invasion [6]–[8], which is not affected by the absence of glycophorin A or B [6] or by antibodies against glycophorin A [7], but it is trypsin sensitive [6], [9], [10]. The putative sialic acid-independent trypsin-sensitive receptor on erythrocytes has been referred to as “X” [9]. The relevance of the sialic acid-independent pathway has been shown by the demonstration that many field isolates can utilize this invasion pathway [11], [12]. Therefore, the identification of the host and parasite molecules involved in this pathway is a necessary step towards the ultimate goal of developing a malaria vaccine that effectively blocks erythrocyte invasion.

We considered CR1 to be a good candidate for being “X” because, like “X”, CR1 is known to be highly sensitive to trypsin and does not contain sialic acid [13]. In addition, CR1 is used by several pathogens as a receptor for entry into host cells [14], [15]. Structurally, it is a 200 kD integral membrane protein found on all erythrocytes and most leukocytes and it is composed of 30 complement control protein (CCP) modules which can be organized, based on their degree of homology, into long homologous repeats (LHR) A–D [16]. It also serves as a co-factor to Factor I for the inactivation of C3b and accelerates the decay of C3 and C5 convertases and thus protects cells from autologous complement attack [17]. CR1 also mediates the binding of mature infected erythrocytes (schizonts) to uninfected erythrocytes (rosetting) [18].

## Results

### Antibodies to CR1 and Soluble CR1 (sCR1) Block Sialic Acid-independent Invasion

**Figure 1:**
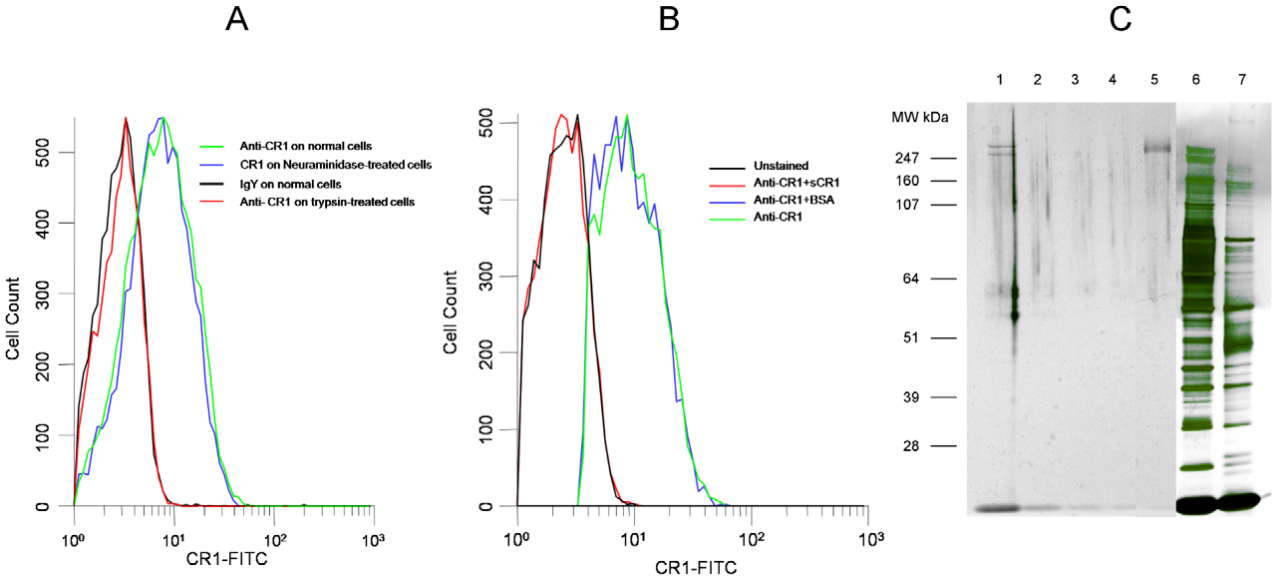
Chicken anti-human CR1 recognizes red cell CR1. (A) Binding of chicken anti-human CR1 Fab is abolished by treatment of erythrocytes with trypsin but not with neuraminidase. (B) Binding of chicken anti-CR1 Fab to human red cells is abolished by incubation with soluble CR1 (sCR1) but not by bovine serum albumin. (C) PAGE of chicken anti-CR1 immunoprecipitates, sCR1 and red cell lysates run on Novex Nupage Bis-tris 4–12% acrylamide gel under reducing conditions and stained with silver. Lane 1: Immunoprecipitate with chicken anti-CR1 from intact red cell lysate. Lane 2: Immunoprecipitate with chicken anti-CR1 from trypsin-treated red cell lysate. Lane 3: Immunoprecipitate with control chicken IgY from intact red cell lysate. Lane 4: Immunoprecipitate with chicken IgY from trypsin-treated red cell lysate. Lane 5: 0.01 µg of purified sCR1. Lane 6: 1 µl of intact red cell lysate used for immunoprecipitation. Lane 7: 1 µl of trypsin-treated red cell lysate used for immunoprecipitation.

**Figure 2:**
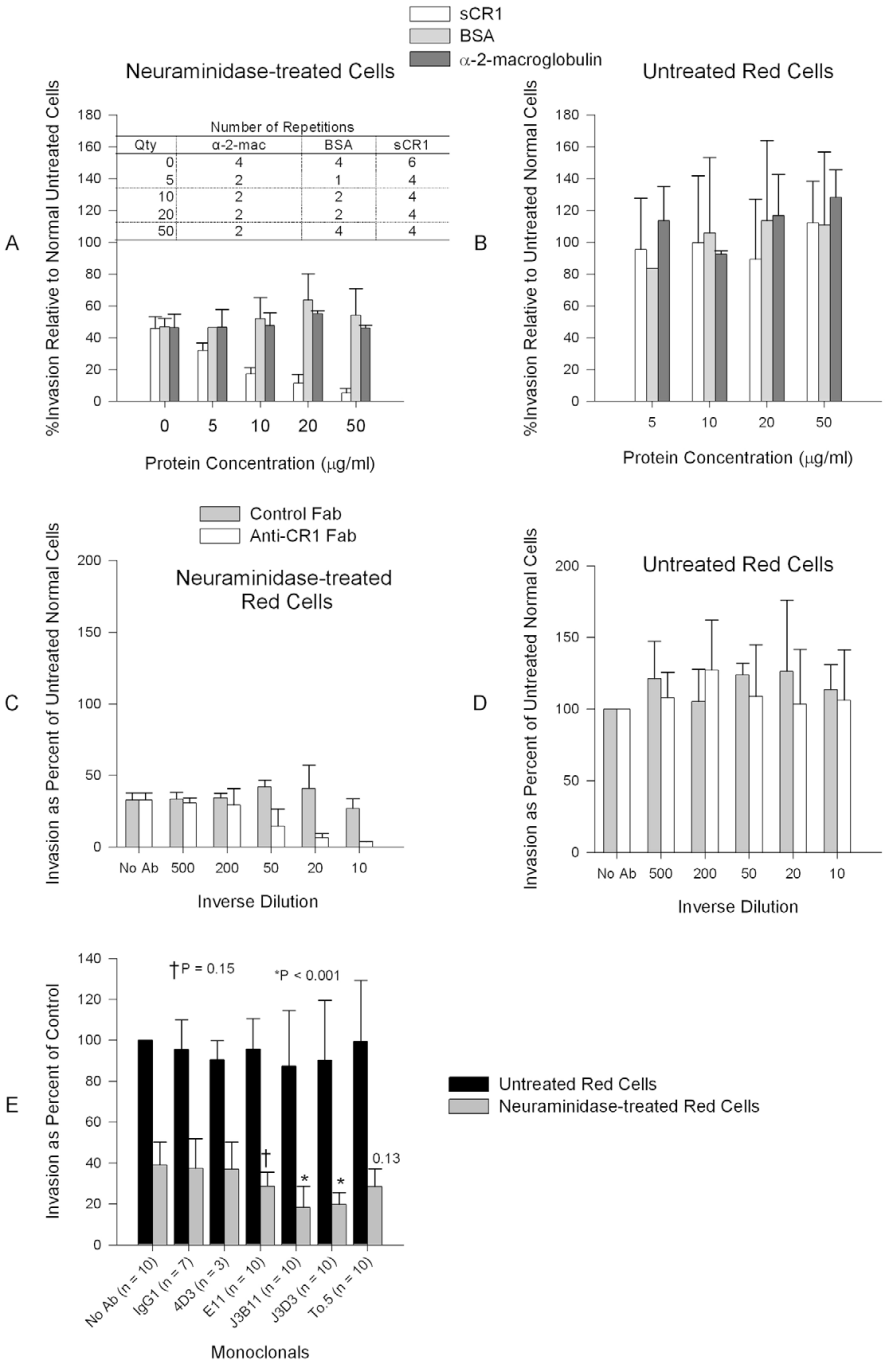
Inhibition of *P. falciparum* 7G8 invasion by sCR1 and antibodies against CR1. (A) sCR1 blocked invasion of neuraminidase-treated erythrocytes in a dose-dependent manner while bovine serum albumin (BSA) and α-2-macroglobulin had no effect. (B) No protein had an effect on the invasion of untreated erythrocytes. (C) Chicken anti-human CR1 Fab blocked invasion of neuraminidase-treated erythrocytes in a dose-dependent manner while purified chicken IgY Fab control had no effect. (D) No antibody had effect on the inhibition of intact red cells. (C–D) The concentration of antibody stocks was 80 µg/ml. Summary of three experiments. (E) Inhibition of *P. falciparum* 7G8 invasion using anti-CR1 monoclonal antibodies. Numbers above bars are P values for the comparison with the no antibody (No Ab) control using Dunnett's pairwise multiple comparison t-test, taking into account matching by experiment date. An IgG_1_ irrelevant monoclonal (R&D Systems, Minneapolis, MN, USA) and anti-CD55 monoclonal (clone NaM16-4D3) (4D3) (Santa Cruz Biotechnology, Inc.) were used as negative controls. E11, To5, J3D3, and J3B11 are anti-CR1 monoclonal antibodies. All monoclonal antibodies were used at 1 µg/ml except in two experiments where every monoclonal was used at 15 µg/ml. Bars represent means ± standard deviations. Invasion rates in untreated controls (no antibody) ranged from 2% to 14%.

In order to test the hypothesis that CR1 is the sialic acid-independent receptor on erythrocytes we used the *P. falciparum* sialic acid-independent laboratory strain 7G8 [6]. We initially used a chicken polyclonal anti-human CR1 that recognizes CR1 on red cells specifically as measured by flow cytometry (Figure 1A and 1B) and is capable of immunoprecipitating CR1 from a red cell lysate (Figure 1C). Incubation of neuraminidase-treated erythrocytes with either sCR1 [19] or anti-human CR1 Fab blocked 7G8 invasion of neuraminidase-treated erythrocytes in a dose-dependent manner (Figure 2A–D) but had no discernible effect on the invasion of untreated erythrocytes. In order to determine whether we were working at excess concentrations of antibodies and sCR1 we measured the invasion inhibition by anti-CR1 antibody and sCR1 under increasing starting parasitemia. We observed stable inhibition of invasion within a wide range of starting parasitemias (Figure S1).

**Figure 3:**
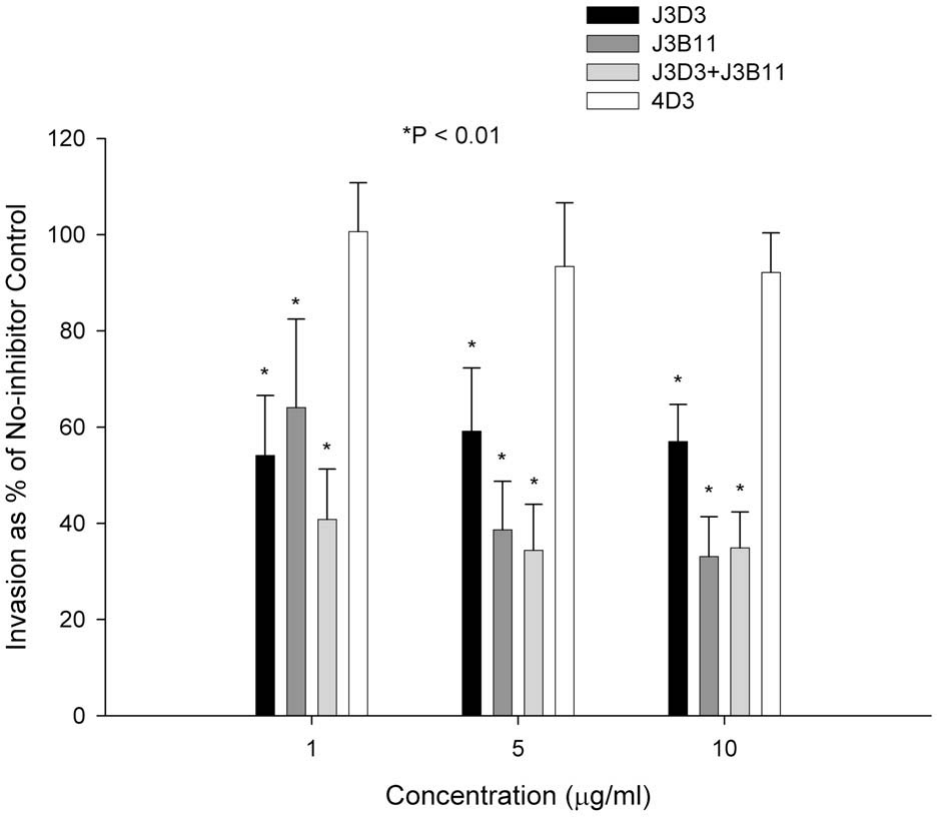
Effect of combined monoclonals on sialic acid-independent invasion of 7G8. Monoclonals J3B11 and J3D3 were used singly or in combination. When used in combination, each monoclonal was used at half of the total concentration. 4D3 is a negative control antibody that recognizes red cell CD55. P values are for the comparison to the no-inhibitor control using Dunnett's pairwise multiple comparison t-test using matching by experiment date. Parasitemia was determined using flow cytometry. The figure represents a summary of 3 experiments. Bars are means ± standard deviations.

Next, to further test the specificity of our findings and narrow down the binding site within CR1 we used a panel of monoclonal antibodies directed against various CR1-defined CCPs: J3D3, E11, To5, and J3B11. J3B11 and J3D3 were the most effective in blocking invasion (Figure 2E). These monoclonal antibodies are known to bind to adjacent CCPs within the C3b binding sites and the CR1 binding site may overlap these two epitopes [20]. J3B11 also interferes with the binding of PfEMP-1 [21], the putative malaria ligand that mediates rosetting and cytoadherence of schizonts to endothelial cells [18], [22]. Although published data suggest that J3D3 and To5 bind within the same group of CCPs [20], the fact that To5 showed minimal, if any, inhibition suggests that finer epitope mapping of this antibody may demonstrate that its epitope is distinct from J3D3. E11 recognizes CCPs that also contain the epitopes for J3D3 and J3B11 but its epitope seems to be more conformationally dependent [20]. To further confirm our results and to look for synergy between J3B11 and J3D3 we repeated these studies using these monoclonals singly or in combination (Figure 3). The results showed that at 1 µg/ml there was some synergistic effect resulting from the combination of antibodies. However, at concentrations of 5 µg/ml or higher J3B11 was more effective than J3D3 and there was no synergy. Therefore, J3B11 appears to be s binding closest to the ligand binding site in CR1.

### CR1 Is a Sialic Acid-independent Receptor for Several Laboratory Strains and Wild Isolates of *P. falciparum*

**Figure 4:**
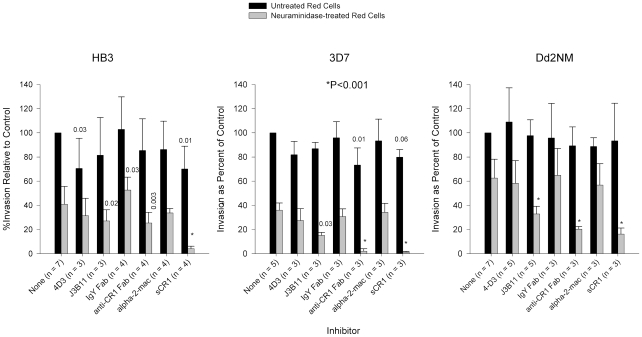
Inhibition of invasion of *P. falciparum* strains HB3, 3D7, and Dd2NM. HB3 invasion of neuraminidase-treated cells was least affected by monoclonal and polyclonal antibodies against CR1. Monoclonal antibodies were used at 4 µg/ml. Fab fragments were used at 8 µg/ml and purified proteins were used at 50 µg/ml. Numbers above bars are P values for the comparison with the no-inhibitor control using Dunnett's pairwise multiple comparison t test, taking into account matching by experiment date. Bars represent means ± standard deviations. Alpha-2-mac = α-2-macroglobulin. Invasion rates for untreated erythrocytes with no inhibitor ranged from 3% to 37%.

In an effort to test whether our results represent a general phenomenon, we examined several additional *P. falciparum* sialic acid-independent laboratory strains (HB3, 3D7, and Dd2NM) [8], [9]. In two of the three laboratory strains (HB3 and 3D7) anti-CR1 Fab or sCR1 resulted in a reduction of invasion of untreated erythrocytes that was statistically significant (Figure 4). Interestingly, the irrelevant anti-CD55 monoclonal antibody 4D3 reduced invasion of untreated erythrocytes by HB3 but had no effect on the other strains. In contrast to untreated erythrocytes, invasion of neuraminidase-treated cells was consistently reduced in all strains by the use of polyclonal or monoclonal antibodies against CR1 as well as by sCR1. Monoclonal and polyclonal antibodies were least effective in blocking HB3 invasion and most effective in blocking 3D7 invasion. sCR1 was usually more effective than antibodies in reducing the sialic acid-independent invasion of red cells (>90% for HB3 and 3D7, and 74% for Dd2NM). Chicken IgY Fab control had a paradoxical effect by increasing the invasion of HB3. Altogether, these results suggest that the use of CR1 as an erythrocyte receptor is common in sialic acid-independent strains, but strains such as HB3 and Dd2NM may utilize additional sialic acid-independent receptors.

In addition to the laboratory strains mentioned above, we examined the role of CR1 in the invasion of red cells in three sialic acid-independent wild strains from Kenya (Table S1). Like the laboratory strains tested, the invasion of neuraminidase-treated red cells was inhibited by sCR1 and chicken anti-CR1. However, unlike laboratory strains, we observed modest but significant inhibition of invasion of intact red cells especially with sCR1.

### Merozoites Interact with Red Cell CR1 and with sCR1-coated Microspheres

**Figure 5:**
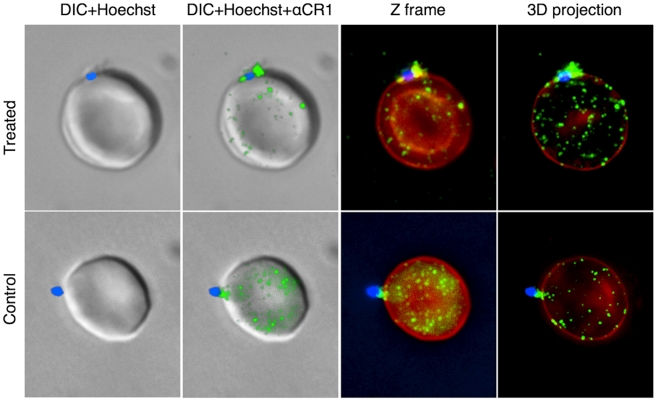
Representative examples of interaction between merozoites and CR1 on neuraminidase-treated and untreated control erythrocytes. CR1 shows a characteristic speckled pattern with aggregation around the merozoite. All negative control antibodies showed no staining. CR1 and glycophorin A were stained with specific antibodies followed by fluorochrome-conjugated secondary antibodies against glycophorin (red) and CR1 (green). Merozoites (blue) were stained with the nucleic acid specific stain (Hoechst 33342). DIC = differential interference contrast.

**Figure 6:**
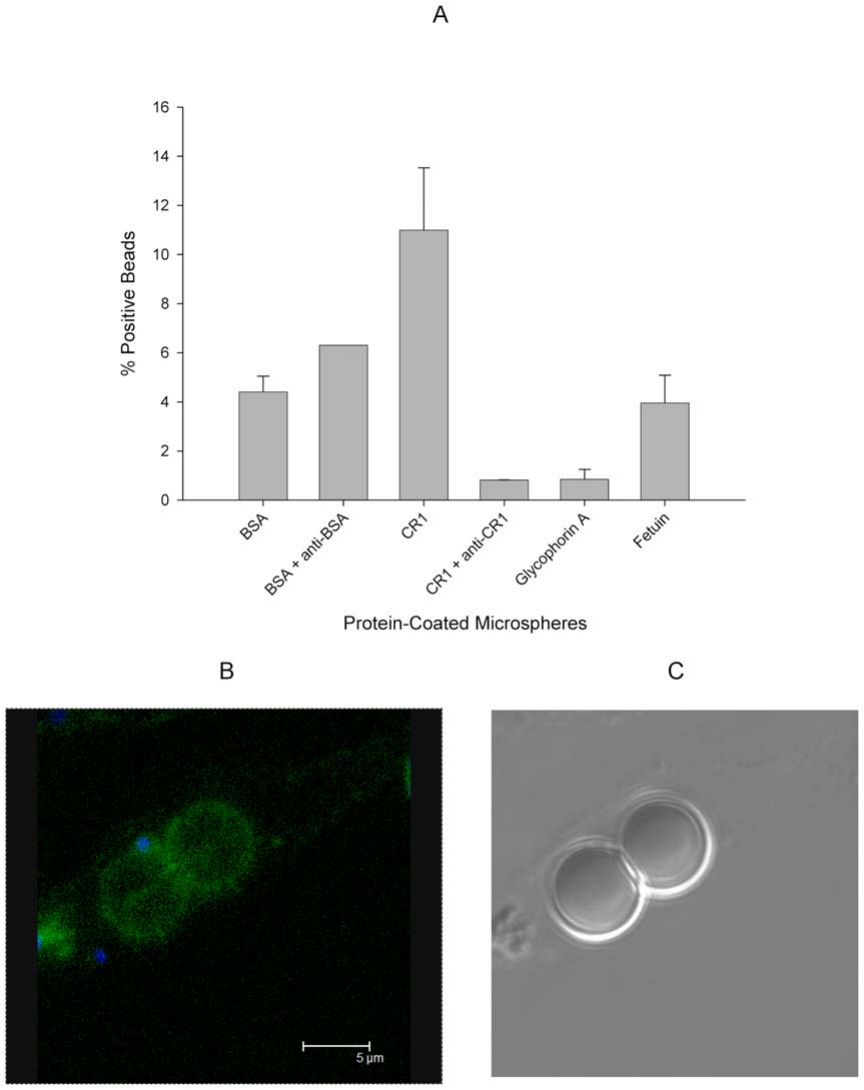
7G8 merozoites bind preferentially to sCR1-coated microspheres. (A) Protein-coated microspheres were incubated overnight with purified late trophozoites and schizonts. Binding was detected by % of spheres positive for Hoechst staining using flow cytometry. Bars represent means ± standard deviations. The figure represents a summary of 2–3 experiments except for the rabbit anti-BSA which was included in one experiment. (B) Fluorescence image of sCR1-coated microspheres showing attached merozoites (blue) and surface sCR1 (green). (C) Differential interference contrast image of microspheres in B.

To demonstrate direct interaction between merozoites and CR1, we performed immunofluorescent microscopy using freshly released merozoites. In agreement with previous observations [23], CR1 appeared as a punctate or speckled pattern on red cells which disappeared upon trypsinization (Figure S2). Merozoites were observed interacting directly with CR1 on erythrocytes both in neuraminidase-treated and untreated control cells (Figure 5, Figure S3, and Supplementary 3D Videos S1, S2, S3, S4, S5, and S6). Although not all merozoites were seen to interact directly with CR1, examples of this interaction were easy to find. When interaction was observed, CR1 staining always appeared to be more intense around the merozoite, suggesting that capping may be occurring. In addition, while the interaction often appeared to be focal, in some instances merozoites seemed to be partially or completely coated by CR1 (Figure S3B and S3D). We observed no clearly discernible difference between 7G8 and the sialic acid-dependent strain Dd2 by immunofluorescent microscopy (data not shown), suggesting that both strains can interact with CR1 or that this technique lacks sensitivity to detect differences in the way the two strains interact with CR1. To further document the interaction of merozoites with CR1, we incubated enriched schizonts and late trophozoites with polystyrene microspheres coated with sCR1, BSA, glycophorin A, or fetuin. Figure 6 shows that merozoites bind to CR1-coated microspheres with greater frequency than to microspheres coated with BSA, glycophorin A, or fetuin. Further, the binding to sCR1-coated microspheres was inhibited by chicken anti-CR1 whereas the binding to BSA-coated microspheres was not inhibited by polyclonal rabbit anti-BSA.

### CR1 Levels Correlate with the Degree of Erythrocyte Invasion

**Figure 7:**
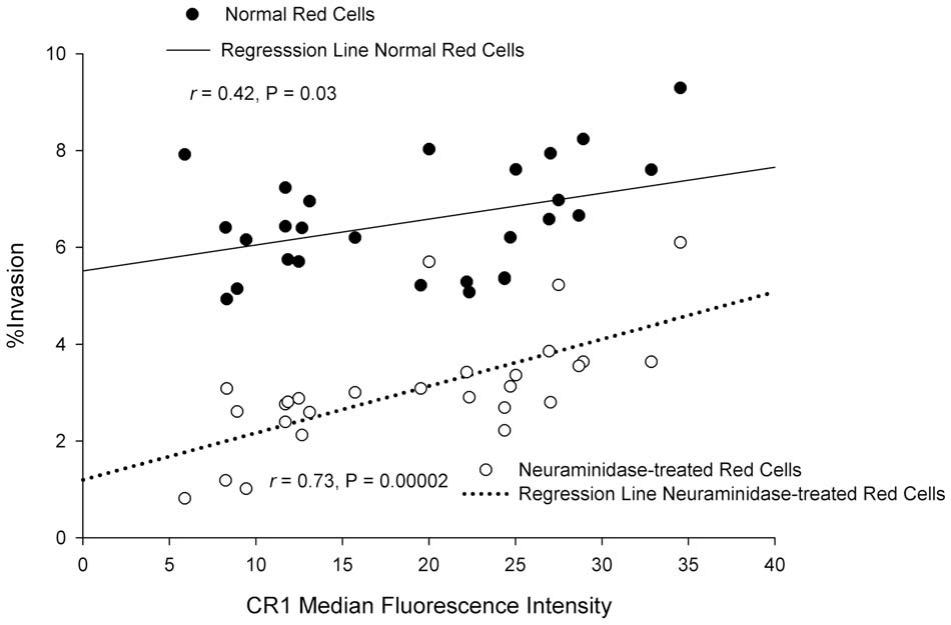
Correlation between CR1 median fluorescence intensity (MFI) and invasion of erythrocytes by *P. falciparum* 7G8. Erythrocytes from 27 healthy donors were used. To control for day-to-day variation the percent invasion as well as the CR1 MFI were normalized to an erythrocyte standard sample that was used in every experiment. The CR1 MFI was re-measured once in 4 samples with extreme values. In 19 donors the invasion assay was repeated 1–3 times. When more than one measurement was done, the mean was used for normalization.

Based on our findings, we predicted a correlation between CR1 expression level and the level of *P. falciparum* invasion, especially in neuraminidase-treated cells. In lieu of the absence of individuals with complete CR1 deficiency, we took advantage of the fact that CR1 is known to have two quantitative alleles (H and L) that are co-dominant and result in the distribution of CR1 levels into low (LL), medium (HL), and high (HH) expressors [16]. We used flow cytometry to determine the CR1 median fluorescence intensity (MFI) of 75 healthy individuals. Erythrocytes of 27 individuals, spanning the range of low, medium, and high expressors, were used for invasion assays. A positive correlation was found between CR1 MFI and invasion for both intact and neuraminidase-treated red cells. Furthermore, this relationship was much stronger in neuraminidase-treated cells than in untreated cells (Figure 7), which is consistent with a greater role of CR1 as a receptor in the absence of sialic acid.

### *P. falciparum* Invades CR1 Transgenic Mouse Red Cells Preferentially Over Wild-type Red Cells

**Figure 8:**
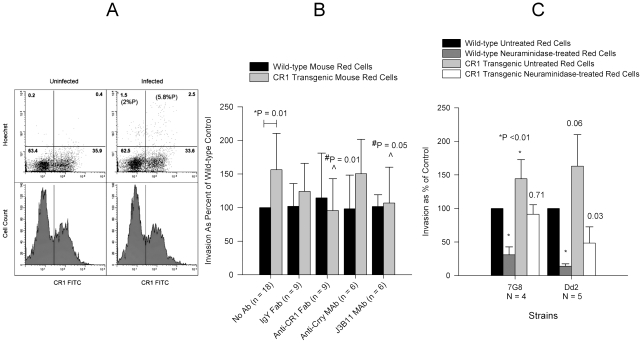
Invasion of wild-type and human CR1 transgenic mouse erythrocytes by *P. falciparum*. (A) 7G8 invaded CR1 transgenic mouse erythrocytes preferentially in a mixed culture of wild-type and CR1 transgenic mouse erythrocytes incubated overnight with or without schizonts and analysed by flow cytometry. Upper panels show dot plots of Hoechst negative (uninfected) and positive (infected) erythrocytes. Numbers in parenthesis represent the percent parasitemia (%P). Numbers without parenthesis in each quadrant represent the percent cells from the total population. The lower panels show CR1 fluorescence histograms and the demarcation between CR1 positive (rectangle) and negative erythrocytes. (B) Effect of polyclonal and monoclonal antibodies on the invasion of wild-type and CR1 transgenic mouse erythrocytes by 7G8. Only significant P values are shown. *P is for the comparison between wild-type and CR1 transgenic red cells with no antibody. #P is for the comparison between antibody and no antibody. Invasion in wild-type erythrocytes ranged from 0.2% to 4.5%. (C) Comparison of invasion of wild-type and CR1 transgenic mouse erythrocytes between 7G8 and Dd2. Numbers above bars represent P values for the comparison to the wild-type untreated control. Invasion was measured by flow cytometry and ranged from 0.45% to 5.5% in wild-type untreated erythrocytes. All bars represent means ± standard deviations.

Finally, we compared the *P. falciparum* invasion of human CR1 transgenic mouse erythrocytes [23] to wild-type mouse erythrocytes. In the mouse, CR1 is expressed mostly on B cells and not on red cells [24]. Although mouse glycophorins contain sialic acid, *P. falciparum* invades mouse erythrocytes at a lower rate than human erythrocytes [25] probably due to structural differences between human and mouse glycophorins [26]. *P. falciparum* 7G8 invaded CR1 transgenic mouse erythrocytes preferentially over wild-type erythrocytes (Figure 8). Anti-CR1 monoclonal J3B11 and polyclonal anti-CR1 Fab reduced the invasion of transgenic erythrocytes by 7G8 down to wild-type levels (Figure 8B). While the sialic acid-dependent strain Dd2 also showed increased invasion of CR1 transgenic mouse erythrocytes, following neuraminidase treatment the invasion decreased dramatically compared to that of 7G8 (Figure 8C). These experiments suggest that both sialic acid-independent and dependent strains are able to interact with CR1, but for the latter CR1-mediated invasion may rely on the presence of intact sialic acid on glycophorin.

### Invasion of Neuraminidase-treated Red Cells by Sialic Acid-dependent Strains Is Less Sensitive to Inhibition by Anti-CR1 and sCR1

**Figure 9:**
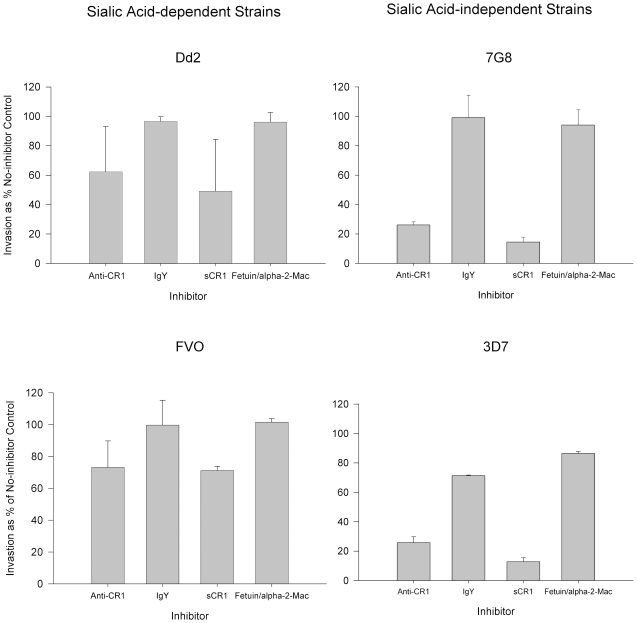
Inhibition of invasion of neuraminidase-treated red cells by sialic acid-dependent and independent strains. Bars represent means ± standard deviations of the invasion as % of the no-inhibitor control. The figure represents a summary of 2–4 experiments. Parasitemias were determined by flow cytometry.

To further explore the interaction of sialic acid-dependent strains with CR1, we compared the ability of anti-CR1 and sCR1 to inhibit the invasion of neuraminidase-treated red cells in sialic acid-dependent strains Dd2 and FVO and sialic acid-independent strains 7G8 and 3D7 (Figure 9). While anti-CR1 and sCR1 inhibited 75–90% of invasion in 3D7 and 7G8, the magnitudes of inhibition in Dd2 and FVO were only 30–50%. Therefore, the invasion of neuraminidase-treated red cells by sialic acid-dependent strains, although minimal, is much less sensitive to inhibition by anti-CR1 and sCR1.

## Discussion

We have shown in a logical and systematic manner that CR1 is a sialic acid-independent receptor for *P. falciparum*. This molecule was previously dismissed when the invasion of erythrocytes expressing very low levels of CR1 (Helgeson phenotype) by sialic acid-independent strain Dd2SNM (similar to Dd2NM used here) was not completely abolished after neuraminidase treatment [27]. These results may be explained by the efficiency of very low levels of CR1 in promoting sialic acid-independent invasion and/or by the presence of an alternative receptor used by Dd2NM (Figure 2). Using the prototypical sialic acid-independent strain 7G8, we demonstrated that sCR1 as well as polyclonal and monoclonal antibodies directed against CR1 drastically reduced or eliminated sialic acid-independent invasion while control proteins and antibodies did not. Similar results were obtained with the sialic acid-independent laboratory strains 3D7, HB3, and Dd2NM. However, for HB3 and Dd2NM CR1 did not appear to be the only sialic acid-independent receptor since these strains showed some residual invasion of neuraminidase-treated red cells in the presence anti-CR1 antibodies and sCR1. In addition, we have shown that the sialic acid-independent invasion of three field isolates from Kenya can also be inhibited by these reagents.

In two of the four laboratory strains tested here we were able to show significant inhibition of intact erythrocytes with anti-CR1 Fab and/or sCR1 ranging from 20–30% and similar results were obtained using wild isolates from Kenya. Although sCR1 is present in serum, its levels are much lower than those required for inhibition of merozoite invasion [28]. These findings suggest that for many malaria strains CR1 is an alternative receptor to glycophorins on intact red cells. The partial inhibition of invasion of intact red cells by anti-CR1 and sCR1 is not surprising given that there are in the order of 10^6^ molecules of glycophorin A per erythrocyte [29] compared to an average of 600 of CR1. The correlation between invasion and CR1 expression level further suggests that the contribution of CR1 to the overall invasion is determined by the number of CR1 molecules present on the red cell. In addition, invasion pathways may work in a hierarchical manner and less dominant pathways only become more prominent in the face of silencing of more dominant ones [30].

Since there is no complete absence of CR1 among humans, to show the effect of complete absence of CR1 on invasion we compared the invasion of wild-type mouse red cells, which do not express CR1, and mouse transgenic red cells expressing human CR1. Using this system we showed that expression of CR1 rendered red cells more susceptible to *P. falciparum* invasion. Interestingly, both sialic acid-independent and dependent strains showed increased invasion of CR1 transgenic mouse red cells but the latter seemed to require the intact sialic acid of glycophorin for optimal CR1-mediated invasion. This is also supported by the finding that invasion of neuraminidase-treated red cells by sialic acid-dependent strains is less sensitive to inhibition by anti-CR1 and sCR1 (Figure 9). One possible explanation for these observations is that the CR1 ligand in sialic acid-dependent strains may require interaction with both CR1 and glycophorin to mediate invasion optimally. The involvement of merozoite ligands in both sialic-dependent and independent invasion has been postulated [31] and our findings are consistent with this concept.

The ability of merozoites to interact with CR1 was shown by immunofluorescence microscopy and without question by the demonstration that merozoites can bind preferentially to sCR1-coated polystyrene microspheres (Figure 6). That this interaction is mediated by a receptor-ligand interaction was demonstrated by the inhibition induced by chicken anti-CR1. By contrast, the interaction of merozoites with BSA-coated microspheres was not inhibited by rabbit anti-BSA. Surprisingly, binding to glycophorin A-coated microspheres was relatively poor, suggesting that glycophorin may be less effective than CR1 in mediating merozoite attachment. Regardless, these results confirm that merozoites can interact directly with CR1.

*P. falciparum* has at its disposal an extensive array of ligands each of which is involved in one or more invasion pathways defined mostly by enzymatic treatment of erythrocytes. The ligands so far identified belong to two major families of proteins: the erythrocyte-binding like (EBL) family [8], which contains EBA-175 [32], and the recently identified reticulocyte-binding like (RBL) family of proteins: PfRh1, PfRh2a, PfRh2b, PfRh3, and PfRh4 [33]. Two studies have reported that the expression of PfRh4 correlates with sialic acid-independent invasion [34], [35]. Antibodies against this protein blocked sialic acid-independent invasion in one study [36] but not in other [37]. In addition, parasite knockouts of PfRh2a and PfRh2b show decreased sialic acid-independent invasion suggesting that these molecules may also be involved in this pathway [31]. Additional work will be needed to determine whether CR1 serves as receptor for any of the PfRh ligands.

There are several scenarios under which the CR1-mediated invasion pathway could become more prominent. One scenario may occur when the parasite encounters red cell or glycophorin variants that lack the receptors for the dominant pathway. This may explain why the CR1-mediated pathway plays such a major role in the invasion of CR1-transgenic mouse red cells. A second situation may involve the development of a natural immune response by the host against the dominant pathway which may drive switching to a less dominant pathway or selection of parasites that use alternative pathways. Already some evidence from the field suggests that this mechanism may actually be at play in endemic populations [38]. Thirdly, and most importantly, immunologic pressure induced by vaccination against the dominant ligands involved in the glycophorin-dependent pathway but not against the CR1-mediated pathway may lead to selection of strains that are more reliant on the latter [39]. Therefore, it is imperative that ligands from all the major invasion pathways be represented in a future malaria blood stage vaccine. The demonstration that CR1 is a sialic acid-independent receptor of *P. falciparum* will facilitate the identification of its ligand(s) and the development of a vaccine that effectively blocks red cell invasion.

## Materials and Methods

### Parasites and Parasite Culture

Strains HB3 and 7G8 were obtained from the Malaria Research and Reference Reagent Resource Center (ATCC, Manasas, VA, USA). Strain 3D7 Oxford was provided by the Walter Reed Army Institute of Research (WRAIR) through the generosity of David Haynes. Dd2NM was derived as described from a Dd2 stock at the WRAIR. Wild strains were obtained from malaria-infected children in western Kenya under approved protocols. Parasite cultures were maintained and synchronized in O+ blood with 10% heat inactivated serum using temperature cycling.

### Enzymatic Treatment of Erythrocytes

Neuraminidase treatment was as described except that erythrocytes at 50% hematocrit in RPMI 1640 were incubated in 250 mU/ml of *Vibrio cholera* neuraminidase (Sigma-Aldrich, St. Louis, MO, USA). For human red cells, the effectiveness of digestion was verified by loss of binding of mouse anti-human glycophorin A/B clone E3 (Sigma-Aldrich) used at a dilution of 1∶3375 with a secondary FITC-conjugated anti-mouse IgG (Sigma-Aldrich) at a dilution of 1∶50.

### Antibodies

Polyclonal chicken anti-CR1 (Accurate Chemical & Scientific Corp., Westbury, NY, USA) and purified chicken IgY (Biomeda, Foster City, CA, USA) were digested with the use of a commercial kit (Thermo Fisher Sicentific, Rockford, IL, USA) to obtain Fab fragments. The final stocks of Fab antibody were adjusted to a concentration of 80 µg/ml. The following IgG_1_ monoclonal antibodies directed against CR1 were also used: J3D3 (Biomeda Corp., Foster City, CA, USA), To5 and E11 (Accurate), and J3B11 (a generous gift of Dr. Jacques Cohen, Hospital Robert Debré, Reims, France). An IgG_1_ irrelevant monoclonal (R&D Systems, Minneapolis, MN, USA) and anti-CD55 monoclonal (clone NaM16-4D3, IgG_1_) (Santa Cruz Biotechnology Inc., Santa Cruz, CA, USA) were used as additional negative controls for human erythrocytes. For experiments with mouse erythrocytes, we used as negative control an IgG_2a_ rat monoclonal antibody directed against the complement receptor 1 related protein Y (Crry) (Becton-Dickinson, San José, CA, USA) found on the surface of mouse erythrocytes.

### CR1 Immunoprecipitation

Human blood was obtained in citrate phosphate dextrose (Sigma-Aldrich) and washed with RPMI to remove the buffy coat. 100 µl of packed red cells was incubated in 1 ml of RPMI 1640 with 500 µg/ml TPCK-treated trypsin (Sigma-Aldrich) for one hour at 37°C and washed ×3 with RPMI by centrifuging at 2,000 rpm for 5 min. Following the last wash, the pellet was resuspended in 1 ml of double deionised water containing protease inhibitor cocktail (Sigma-Aldrich) and allowed to sit in ice for 30 min. The ghosts were recovered by centrifugation at 10,000 rpm for 10 min and the pellet was washed twice more with deionised water. Finally, the pellet was solubilized with 200 µl of IP lysis buffer (Thermo Fisher Scientific) containing protease inhibitor cocktail (Sigma-Aldrich) and allowed to sit in ice for 30 min after which it was stored at −20°C until used. An additional 100 µl of packed red cells was similarly lysed and solubilized without prior enzyme treatment. Aproximately 100 µg of polyclonal chicken anti-CR1 or chicken IgY were covalently linked to each of two 100 µl volumes of AminoLink Plus Coupling Resin (50% slurry) following the manufacturer's instructions (Thermo Fisher Scientific). 500 µl of IP lysis buffer containing 5 µl of lysate from trypsin-treated or untreated red cells in the presence of protease inhibitor cocktail was added to each chicken anti-CR1 and IgY-linked resin pellets followed by incubation at room temperature for two hours with constant mixing and overnight at 4°C. The following day, the resins were washed exhaustively with IP lysis buffer and the trapped proteins were eluted with 50 µl low pH elution buffer followed by neutralization with 5 µl of 2 M Tris. 10 µl of each eluate was loaded onto separate lanes of a 4–12% Nupage-Novex Bis-Tris denaturing gel (Invitrogen Corp., Carlsbad, CA, USA) followed by silver staining (Thermo Fisher Scientific).

### Invasion Assays

Schizont-infected erythrocytes were separated from uninfected erythrocytes by magnet-activated cell sorting (MACS; Miltenyi BioTec, Bergisch Gladbach, Germany) using “LS” columns. The magnetized columns were equilibrated with 3 ml of RPMI 1640+0.2% sodium bicarbonate (elution buffer). Typically, a 24 ml culture was processed in three 8 ml aliquots. After loading each 8 ml of culture onto the magnetized column, it was washed and the infected erythrocytes eluted after demagnetization with 3 ml of elution buffer each time. The total infected erythrocytes eluted were pelleted and resuspended in complete medium (RPMI 1640, 10% O+ plasma, 0.2% NaHCO_3_, 25 mM Hepes). O+ human erythrocytes, wild-type C57BL/6 mouse erythrocytes, or CR1 transgenic mouse erythrocytes in C57BL/6 background, were plated in duplicate wells of a 96-well plate at a hematocrit of 2–4% in complete medium and were inoculated with schizont-infected erythrocytes to achieve a parasitemia of 0.3 to 5% for human red cells and 4–7% for mouse red cells. Plasma inactivated by heating at 56°C for 45 min was used in most experiments although we have not observed a difference in invasion or invasion inhibition between heat-inactivated and non-inactivated plasma (data not shown). For microscopic readout human red cell invasion, only experiments in which the control untreated erythrocytes gave 2% or higher parasitemia were included in the analysis. Because much lower invasion rates were seen with mouse red cells, all levels of parasitemias were accepted. Blocking antibodies were added to uninfected erythrocytes prior to the addition of schizont-infected erythrocytes. For blocking with soluble CR1 (sCR1) (AVANT Immunotherapeutics, Inc., Needham, MA, USA), bovine serum albumin, fetuin, and α-2-macroglobulin (Sigma-Aldrich) were used as negative control proteins depending on their availability. After a 16–22-hour incubation the ring stage parasites in 1,000 erythrocytes were counted in thin smears stained with Giemsa. The microscopist was always blinded to the experimental group designation of each slide. In some experiments, parasitemia was measured by flow cytometry (see below). When comparing invasion across different donors, the parasitemia was normalized to the parasitemia of a single erythrocyte donor used as control in each assay using the formula 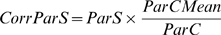, where “CorrParS” is the corrected parasitemia of the sample, “ParS” is the uncorrected parasitemia of the sample, “ParCMean” is the mean parasitemia of all the control samples, and “ParC” is the uncorrected parasitemia of the control for that sample.

### CR1 Flow Cytometry

For detection of CR1, a 4% hematocrit cell suspension was incubated with chicken anti-human CR1 Fab at 8 µg/ml for 30 min at RT and washed three times with RPMI 1640. The cell sample was incubated with a 1∶50 dilution of FITC-conjugated goat anti-chicken IgG (Sigma-Aldrich) for 30 min at RT and washed three times with RPMI and stored in 1% paraformaldehyde at 4°C until acquisition. During acquisition, erythrocytes were gated on the basis of their forward and side scatter characteristics and the median fluorescence intensity (MFI) was measured using logarithmic amplification. To correct for day-to-day variation when comparing the CR1 MFI among a series of donors, the MFI was normalized to the mean of a control sample that was included in all the assays using the formula 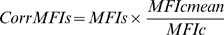, where “CorrMFIs” and “MFIs” are the corrected and uncorrected sample MFI respectively, “MFIcmean” is the mean of all the MFI values of the standard control, and “MFIc” is the mean of the control obtained in parallel with the sample.

### Detection of Parasitized Red Cells by Flow Cytometry

For detection of parasitized erythrocytes, cells were incubated in 1 µg/ml Hoechst 33342 (Invitrogen) prior to fixation. The background staining of an uninfected red cell sample was always subtracted. Mouse red cells were differentiated from human red cells by use of PE-Cy5-labeled rat anti-mouse glycophorin (clone TER-119, Becton-Dickinson, San José, CA, USA) at a dilution of 1∶100. At least 10,000 erythrocytes were acquired for each sample. Acquisition was done using a LSRII flow cytometer (Becton-Dickinson) equipped with a UV laser and analysis was done using Winlist v5.0 (Verity Software, Topsham, ME, USA). For determination of % parasitemia (%P) of mouse red cells using flow cytometry we used the following formula
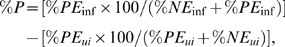
where %PE_inf_ = %Hoechst positive erythrocytes in malaria culture, %NE_inf_ = %Hoechst negative erythrocytes in malaria culture, %PE_ui_ = % Hoechst positive erythrocytes in uninfected culture, and %NE_ui_ = % Negative erythrocytes in uninfected culture. We always observed good correlation between flow cytometry results and microscopy.

### Polystyrene Microsphere Merozoite Binding Assay

Proteins were linked to 6 µm carboxylated polystyrene microspheres using the PolyLink coupling kit (Polyciences Inc., Warrington, PA, USA). 10 µg of each protein was incubated overnight at room temperature with 50 µl of a 2.6% suspension of activated microspheres. The following day, the microspheres were washed thrice with wash buffer by centrifugation at 1,000×g for 5 min. The microspheres were then resuspended in PBS containing 100 µg/ml of BSA and incubated at room temperature for 1 hr to block any remaining binding sites. Finally, the microspheres were washed thrice again in PBS and stored at 4°C in 500 µl of PBS until used. Coating of microspheres with proteins was confirmed by flow cytometry using polyclonal antibodies against each protein (data not shown). 7G8 late trophozoites and schizonts were purified by Percoll gradient centrifugation [40]. Approximately 1×10^6^ infected red cells (>80% parasitemia) were incubated with 8×10^5^ microspheres in duplicate wells of a 96-well tissue culture plate containing 120 µl of complete medium and 25 µg/ml gentamicin. Chicken polyclonal anti-CR1 (10 µl of 1 µg/ml in PBS) or rabbit anti-BSA (Sigma-Aldrich) (5 µl of 20 µg/ml in PBS) was added to separate duplicate wells. The plate was then incubated overnight at 37°C in a sealed gas impermeable bag containing 5% CO_2_, 5% O_2_, and 90% N_2_. The following day, Giemsa-stained smears of each well were prepared to confirm merozoite release and attachment. An equal volume of PBS 2% paraformaldehyde containing 4 µg/ml Hoechst 33342 (Invitrogen Corp.) was added to each well and the plate was then stored at 4°C until acquisition took place. Acquisition was carried out on an LSR II (Becton-Dickinson) using the violet and 488 laser lines (Pennsylvania State University College of Medicine Flow Core (www.hmc.psu.edu/core/flow/overview.htm). The microspheres were gated based on their forward and side scatter characteristics using logarithmic amplification. The % microspheres that showed Hoechst staining was determined.

### Immunofluorescent Microscopy of Polystyrene Microspheres

Following overnight culture of malaria-infected red cells with sCR1 or BSA-coated microspheres, a 50 µl aliquot of each culture was centrifuged at 14,000 rpm for a few seconds and resuspended in 1∶100 chicken anti-CR1 in complete culture medium and incubated at room temperature for two hours. The microspheres were then washed twice with PBS and resuspended in 1∶100 DyLight 488-labeled goat anti-chicken IgY (KPL, Inc., Gaithersburg, MD, USA) in PBS and incubated for 2 hours at room temperature. After one wash in culture medium, the pellet was resuspended in 2% paraformaldehyde with 4 µg/ml of Hoechst 33342 (Invitrogen) and incubated overnight at 4°C. The following day, 5 µl of microsphere suspension was spotted onto a slide, dried, and fixed with methanol. The microspheres were observed under a Leica TCS SP2 AOBS confocal microscope using the 405 and 488 laser lines (www.hmc.psu.edu/core/microscopy/confocal.htm).

### Red Cell Immunofluorescent Microscopy

Schizont-infected erythrocytes were incubated with 10 µg/ml of leupeptin (Sigma-Aldrich) for 8–12 hrs in complete medium at 37 °C, and then washed three times by centrifugation (400×g/4 min) and resuspension in RPMI 1640 (Sigma-Aldrich) containing 25 mM Hepes and 0.2% NaHCO_3_. Fresh uninfected erythrocytes, normal or neuraminidase-treated, were then added to the parasitized erythrocytes to a final hematocrit of 2% and parasitemia of 2% in complete medium and allowed to incubate for 30–60 minutes until at least 50% of the parasite population were membrane-enclosed merozoites. At this point, they were diluted to 0.2% hematocrit and added to 14 mm collagen-coated No.1 coverslips (MatTek Corporation, Ashland, MA, USA). After incubating for 30 min at 37 °C to allow for attachment of the erythrocytes to the collagen, the cells were fixed and prepared for immunofluorescence using methods described by Tokumasu and Dvorak [41]. Briefly, erythrocytes were cross-linked with 50 mM dimethyl suberimidate dihydrochloride (DMS) (Thermo Fisher Scientific) in a buffer containing 100 mM sodium borate buffer (pH 9.5) and 1 mM MgCl_2_ for 1 hr at RT, followed by fixation with 1% paraformaldehyde in PBS for 1 hr at RT. DMS and paraformaldehyde were quenched by incubating with 0.1 M glycine in PBS, pH 7.4, for 1 hr at RT. After washing in PBS for 5 min, the erythrocytes were blocked with 3% BSA in PBS with 0.2% Tween 20 (blocking buffer) for 30 min at RT. Chicken polyclonal anti-CR1 Fab (8 µg/ml), and rabbit polyclonal anti-glycophorin A IgG (Accurate) (diluted 1∶10 for neuraminidase-treated cells and 1∶50 for normal cells) were added in blocking buffer for 1 hr at RT. Following three washes with PBS with 0.2% Tween 20, the secondary antibodies goat anti-chicken IgG-Alexa 488, and goat anti-rabbit IgG-Alexa 555 (Santa Cruz) were diluted 1∶50 in blocking buffer and incubated for 30 min at RT. Nuclei were stained with Hoechst 33342 (2 µg/ml in PBS) (Invitrogen) for 15 min at RT. After a final wash in PBS with 0.2% Tween 20, the coverslips were dried, mounted with Antifade (Biomeda), and sealed to standard microscope slides. The fluorescence z-series were collected on a LEICA DM RXA fluorescence microscope at 100× under oil immersion. Prior to reconstruction, the images were unsharp masked to remove the background and blurry parts of the images and, subsequently, contrast enhanced using TIFFany3 image processing software (Caffeinesoft, Inc., www.caffeinesoft.com). This method provides highly satisfactory results for small sharp features in the images as verified by comparison to the original data sets. The 3D image reconstructions from 40-plane image stacks were performed with BICViewer (Bioinstrumentation Center, USUHS, Bethesda, Maryland, USA).

### Collection of Mouse Blood

Blood from human CR1 transgenic and wild-type mice in C57BL/6 background was obtained by cardiac puncture following deep anesthesia with inhaled isoflurane. Cardiac blood was anticoagulated with citrate phosphate dextrose, shipped overnight from the University of Massachusetts in Worcester, Massachusetts, to the Walter Reed Army Institute of Research in Silver Spring, Maryland, and used immediately upon arrival.

### Ethics Statement

Collection of human blood samples for this study was conducted according to the principles expressed in the Declaration of Helsinki and under protocols approved by the Human Use Research Committee of the Walter Reed Army Institute of Research and/or the National Ethics Review Committee of the Kenya Medical Research Institute. All patients provided written informed consent for the collection of samples and subsequent analysis. All animals were handled in strict accordance with good animal practice as defined by the National Institutes of Health and the Association for the Assessment and Accreditation of Laboratory Animal Care International (AAALAC) under a protocol approved by the University of Massachusetts Medical School Institutional Animal Care and Use Committee.

### Statistical Analysis

Analysis was done using SPSS v11.5 (SPSS Inc., Chicago, IL, USA). The general linear model, an analysis of variance procedure, was used to test for equality of means across several experimental groups taking into account matching across groups by date of assay. If the overall F test was significant, Dunnett's pairwise multiple comparison t-test was used to compare each experimental group mean to the control mean. Spearman rank correlation was used to study the relationship between CR1 level and invasion. The paired samples t-test or the non-parametric Wilcoxon signed rank test, for small samples, was used for the comparison of two paired samples. All tests were 2-sided with α≤0.05.

## Supporting Information

Figure S1 Invasion inhibition by anti-CR1 and sCR1 remains stable over a wide range of parasitemia. Enriched trophozoites/schizonts were added to 2% hematocrit culture at varying concentrations in duplicate wells of a 96-well plate while maintaining the concentrations of inhibitors constant. Invasion rate in the presence of inhibitors (shown on the y axis on the right) was normalized to the no-inhibitor control invasion (shown on the y axis on the left). Inhibition remains constant within the range of starting parasitemias used in this study (up to 5%). Dots represent means ± standard deviations.
(0.05 MB DOC)

Figure S2 Speckled pattern of CR1 in intact red cells is eliminated by treatment with trypsin. (A) Cross-section of intact red cells, (B) Cross-section of trypsinized red cells.
(0.28 MB DOC)

Figure S3 More representative examples of interaction between merozoites and CR1 on the surface of treated and untreated (control) red cells. Merozoites (blue), CR1 (green), and glycophorin A (red). DIC = Differential interference contrast.
(1.96 MB DOC)

Table S1 Effect of Anti-CR1 and sCR1 on invasion of *P. falciparum* wild strains.
(0.04 MB DOC)

Video S1 Merozoite (blue) attached to CR1 (green) on the surface of a red cell. This video corresponds to control in Figure 4, main text.
(1.15 MB MOV)

Video S2 Merozoite (blue)on the surface of a neuraminidase-treated red cell with aggregation of CR1 (green) around the merozoite. This video corresponds to neuraminidase-treated panel in Figure 4 of the main text.
(1.30 MB MOV)

Video S3 Merozoite (blue) attached to the surface of a neuraminidase-treated red cell with aggregation of CR1 (green)between the merozoite and the red cell. This video corresponds to supplementary Figure S1A.
(2.04 MB MOV)

Video S4 Merozoite (blue) attached to the surface of a neuraminidase-treated red cell with aggregation of CR1 (green) around the merozoite. This video corresponds to supplementary Figure S1B.
(0.63 MB MOV)

Video S5 Merozoite (blue) on the surface of an untreated red cell with increased CR1 (green) intensity at one end of the merozoite. This video corresponds to supplementary Figure S1C.
(0.52 MB MOV)

Video S6 Merozoite (blue) on the surface of an untreated red cell with increased CR1 (green) intensity around the merozoite. This video corresponds to supplementary Figure S1D.
(1.20 MB MOV)
