# Supplementary material for: Complement Receptor 1 Is a Sialic Acid-Independent Erythrocyte Receptor of Plasmodium falciparum
Source: PLoS Pathog. 2010 Jun 17;6(6):e1000968. doi: 10.1371/journal.ppat.1000968 (PMC2887475; doi:10.1371/journal.ppat.1000968)
Supplement: Figure S1 — Invasion inhibition by anti-CR1 and sCR1 remains stable over a wide range of parasitemia. Enriched trophozoites/schizonts were added to 2% hematocrit culture at varying concentrations in duplicate wells of a 96-well plate while maintaining the concentrations of inhibitors constant. Invasion rate in the presence of inhibitors (shown on the y axis on the right) was normalized to the no-inhibitor control invasion (shown on the y axis on the left). Inhibition remains constant within the range of starting parasitemias used in this study (up to 5%). Dots represent means ± standard deviations. (0.05 MB DOC) [file ppat.1000968.s001.doc]

**Figure S1. Invasion inhibition by anti-CR1 and sCR1 remains stable over a wide range of parasitemia.** Enriched trophozoites/schizonts were added to 2% hematocrit culture at varying concentrations in duplicate wells of a 96-well plate while maintaining the concentrations of inhibitors constant. Invasion rate in the presence of inhibitors (shown on the y axis on the right) was normalized to the no-inhibitor control invasion (shown on the y axis on the left). Inhibition remains constant within the range of starting parasitemias used in this study (up to 5%). Dots represent means ± standard deviations.
