# Supplementary material for: Complement Receptor 1 Is a Sialic Acid-Independent Erythrocyte Receptor of Plasmodium falciparum
Source: PLoS Pathog. 2010 Jun 17;6(6):e1000968. doi: 10.1371/journal.ppat.1000968 (PMC2887475; doi:10.1371/journal.ppat.1000968)
Supplement: Figure S2 — Speckled pattern of CR1 in intact red cells is eliminated by treatment with trypsin. (A) Cross-section of intact red cells, (B) Cross-section of trypsinized red cells. (0.28 MB DOC) [file ppat.1000968.s002.doc]

| 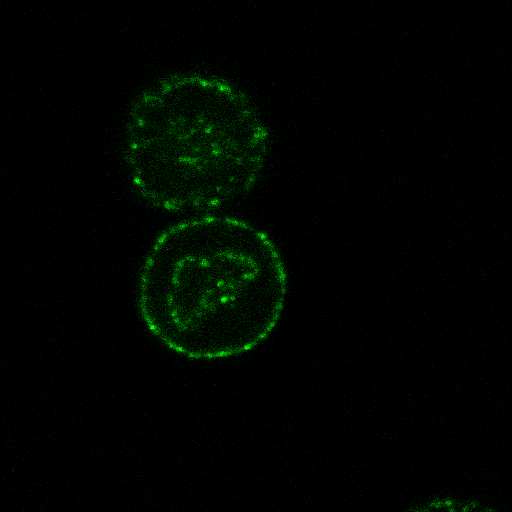A | 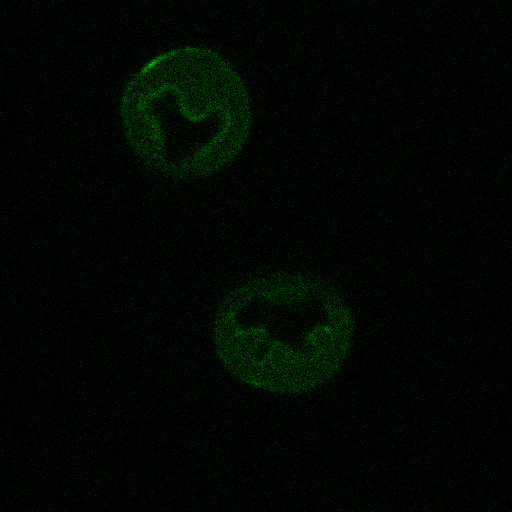B |
| --- | --- |

**Figure S2. Speckled pattern of CR1 in intact red cells is eliminated by treatment with trypsin.** (A) Cross-section of intact red cells, (B) Cross-section of trypsinized red cells.
