# Supplementary material for: Complement Receptor 1 Is a Sialic Acid-Independent Erythrocyte Receptor of Plasmodium falciparum
Source: PLoS Pathog. 2010 Jun 17;6(6):e1000968. doi: 10.1371/journal.ppat.1000968 (PMC2887475; doi:10.1371/journal.ppat.1000968)
Supplement: Figure S3 — More representative examples of interaction between merozoites and CR1 on the surface of treated and untreated (control) red cells. Merozoites (blue), CR1 (green), and glycophorin A (red). DIC = Differential interference contrast. (1.96 MB DOC) [file ppat.1000968.s003.doc]

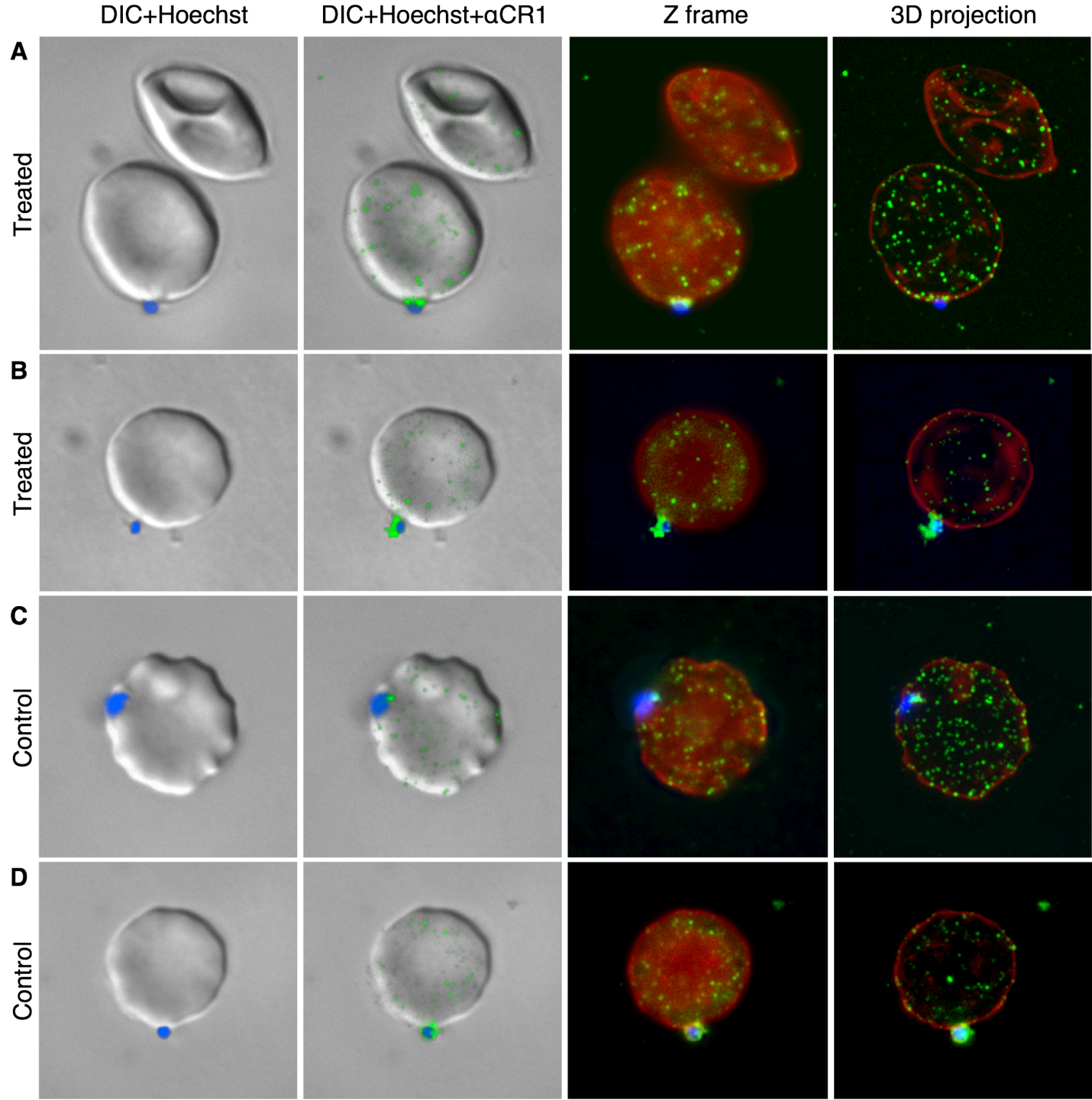


**Figure S3. Additional examples of interaction of merozoites with CR1 on neuraminidase-treated and untreated (control) red cells.**
