# Supplementary material for: Complement Receptor 1 Is a Sialic Acid-Independent Erythrocyte Receptor of Plasmodium falciparum
Source: PLoS Pathog. 2010 Jun 17;6(6):e1000968. doi: 10.1371/journal.ppat.1000968 (PMC2887475; doi:10.1371/journal.ppat.1000968)
Supplement: Table S1 — Effect of Anti-CR1 and sCR1 on invasion of P. falciparum wild strains. (0.04 MB DOC) [file ppat.1000968.s004.doc]

**Table S1. Effect of Anti-CR1 and sCR1 on invasion of *P. falciparum* wild strains.**

|  | Intact Red Cells | | | | Neuraminidase-treated Red Cells | | | | |
| --- | --- | --- | --- | --- | --- | --- | --- | --- | --- |
| Wild Strain | Anti-CR1 | IgY | sCR1 | α-2-Mac/  Fetuin | No Inhibitor | Anti-CR1 | IgY | sCR1 | α-2-Mac/  Fetuin |
| JASC 8-19 (n = 5) | 82.6(6.7)* | 93.1(4.6) | 73.7(7.1)* | 100.2(3.4) | 45.2(2.2) | 10.8(1.6)* | 43.1(2.8) | 11.3(1.8)* | 44.0(3.7) |
| SA005 (n = 3) | 90.2(2.4)* | 93.5(5.9) | 69.4(2.1)* | 92.9(3.8) | 42.8(7.3) | 11.4(1.7)* | 38.3(2.6) | 8.2(1.8)* | 38.4(5.7) |
| SA222 (n = 5) | 84.7(7.8) | 97.4(4.5) | 64.9(3.9)* | 100.9(2.4) | 36.8(2.7) | 13.2(0.7)* | 37.9(4.7) | 14.0(2.4)* | 34.4(3.9) |

Invasion of untreated or neuraminidase-treated red cells in the presence of chicken anti-CR1, IgY, sCR1, alpha-2-macroglobulin (α-2-Mac) or fetuin were expressed as a percentage of invasion of untreated red cells with no inhibitor. Data are presented as means (STD) of three to five separate experiments for each parasite strain, and “*” indicates that invasion inhibition was statistically significant (P<0.02 vs no-inhibitor control for either intact or neuraminidase-treated red cells, Dunnett’s test for multiple comparison with matching).
